# 

**Published:** 1849-10-01

**Authors:** 


### BOOKS AND JOURNALS RECEIVED FOR REVIEW.

Sixth Report of the Managers of the State Lunatic Asylum, U. S., America, 1849, (will be reviewed in the next number.)

Health of Towns; a Digest of several Reports on Sanitary Reform, by W. Simpson, Esq., Surgeon.

Hospital Reports and other Documents, from the German.

State of the Lincoln Lunatic Asylum, 1848.

Report of the Pennsylvanian Hospital for the Insane, by W. Kirkbridge, M.D., for 1848. Philadelphia, 1849.

Proceedings of the Westminster Medical Society, Sess. 1848-9.

A Physician's Holiday, by J. Forbes, M.D., 1849.

Evangile Populaire, par le Dr. Scipon Pinel; Paris, 1849.

Recherches sur la Paralysie Générale Progressive, &c.; par le Dr. L. Lunier, Paris, 1849.

A Remonstrance to the Lord Chief Baron, touching the case of Nottidge *v.* Ripley, by J. Conolly, M.D. Third edition, Lond. 1849.

Thése pour Le Doctorat en Médecine, par Antoine Emile Blanche, du Cathéterisme œsophagien chez les Aliénés. Paris, 1848.

The Morningside Mirror.

Manx Sun, (2 copies.)

On Hip Joint Disease, by W. C. Hugman, Surgeon. Lond. 1849. (An able and useful treatise.)

On Certainty in Medicine, by E. Bartlett, M.D. New York, 1848. (A valuable contribution to medical science.)

On the Pathology of Croup, by Horace Green, A.M., M.D., New York. John Wiley, New York, 1849.

Report of the County Lunatic Asylum, Gloucester, 1849.

Fifty-second Report of the Institution, near York, called the Retreat, for Insane Persons, 1848.

Report of the Kent Lunatic Asylum, 1849.

Report of the Dorset County Lunatic Asylum, 1849.

Report of the Lunatic Asylum for the North and East Riding of Yorkshire, 1849.

Twenty-eighth Annual Report of the Dundee Asylum, 1849.

---

### JOURNALS IN EXCHANGE.

Provincial, Medical, and Surgical Journal. Regularly.
The Zoist. Regularly.
The Dublin Medical Press. Regularly.
The American Journal of Insanity. Regularly.
The Massachusetts Quarterly Review. America. Regularly.
The London Journal of Medicine. London: Taylor. Regularly.
The Monthly Journal and Retrospect of the Medical Sciences. Edinburgh and London. Regularly.
Dr. Ranking's Half-Yearly Abstract of the Medical Sciences. Regularly.
The New York Journal of Medicine and the Collateral Sciences, (July No. just received.) Regularly.
The Transactions of the Provincial, Medical, and Surgical Association. Vol. XVI., Part II. London: J. Churchill.
Nos. XXXIV. & XXXV. of the American Journal of Medical Science. Edited by Isaac Hays, M.D. Philadelphia, 1849. An able periodical. Will be noticed in our next.

---

*** Press of matter has compelled us to defer the publication of several articles of interest and value. Among these is a lengthy analysis of the Reports of the British County Lunatic Asylums, which will certainly appear in the next number of the Journal, as well as notices of most of the works received. Parties desiring an exchange of journals may effect their object by applying to the publisher.

*Full Price given for copies of* No. II. *of the "Journal of Psychological Medicine."*

---

### INDEX FOR VOL. I. & II.

Some of our correspondents have complained of the Index to Vol. I. of the Journal not being sufficiently copious. With the view of remedying this alleged defect, we have had prepared a more full analysis of the volume, which we publish conjointly with the Index for Vol. II.

### INDEX TO VOL. II.

Abercrombie, on phantasms of the brain, 275.
Abuse, self, apology for, offered by Lallemand, 16.
Absurd and dangerous fancies of the dark ages, 220, 234, 240.
Accommodation for lunatics capable of paying a small sum, in England, 394.
——— similar deficiency in Ireland, 394.
Affection, disappointed, frequent cause of insane condition, 213.
Affusion, cold, fatal results from, 165.
Age, present, matter of fact character of, 522.
Agents, supernatural belief in, 224.
Albumen, quantity of, in blood in neuroses, 122.
Allegory of death, 89.
Aldehyde, 170.
America, insanity in, 398.
——— northern states, ratio of mortality in, from insanity, 411.
Anatomy, pathological, and chymistry, resemblances and differences between, 126.
——— morbid, of mental diseases, 483.
Ancients, pantheism of, 61.
Anecdote of Sale, 30.
——— Taylor, 30.
Anxiety, a cause of insanity, 214.
——— Dr. Wigan on, 507—510.
Apothegm of De La Rive, of Geneva, on lunacy, 246.
Appointments, 348.
Apology for Swift's conduct to Varina and Vanessa, 350.
——— for life of Dean Swift, 349.
——— an, for the nerves, 90.
——— discursive nature of the work, 92.
Arc, Jeanne d', reflections on, 225.
Artois, general charge of sorcery against, 228.
Ascarides, cause of self-abuse in children, 26.
Ashley, Lord, letter explanatory of the duties of the commissioners in lunacy, 608.
Association of nerves, 306.
Asylums, lunatic, of Italy, 136.
——— of Nantes, 147.
——— Bloomingdale, admission of cases of delirium tremens, 193.
——— admissions, and re-admissions of all cases in, 197, 198.
——— ages of patients in, 200.
——— admissions arranged according to decennial periods, 200.
——— lunatic, in America, 398.
——— New York, state lunatic asylum, interesting report of, 405.
——— travelling commission of, 413.
——— new county, for Middlesex, at Colney Hatch, foundation of, 491.
——— Hanwell lunatic, fourth report of, 418
Aubanel on false membranes of arachnoid, 128.

Bandages, tight, dangerous to functions of brain, 37.
——— not confined to lower orders amongst the French, 31.
Bandaging the head in early life, bad effects of, 30.
——— ultimate bad results from, 31.
——— erroneous views of those who deny effect of external pressure, 33.
Barclay, on life and organization, 59.
Beatty, Dr., on persons found dead, 79.
Bed, new invented, by Aubanel, for the insane, 141.
Benvenuto Cellini, illusions of, 272.
Bethlehem, restraint of insane falling gradually into disuse, 257.

Bibliotheque du Medicin Practicien. Tome neuvième, traité de maladies du cerveau, maladies mentales, maladies nerveuses, 534.
Blisters, mercurial, in encephalitis, 169.
Blood, the ostensible site of the vital principle, 75.
Bloodletting, dangerous, if too frequent in general palsy of the insane, 346.
Blood, on, in the neuroses, by Dr. Michea, 118.
——— fibrine, proportion of, in the neuroses, 122.
——— albumen, proportion of, 122.
——— serum, proportion of, 122.
——— of the insane, Dr. Erlenmeyer, on the, 139.
——— pressure on a nerve, effects of, according to Bichat, 91.
——————— according to Swan, 91.
——— and innervation, mutual relation of, 93.
Bloomingdale Asylum, New York, history and statistics of, 189.
Blumenbach, his *nisus formativus*, 73.
Boismont de Brierre, on the classification of mental diseases, 551.
Books received, 188, 348, 635.
Bory de St. Vincent, singular observations on vegeto-animal forms, 71.
Brain, sole centre of human nervous system, 302.
—— softening of, by Dr. Winslow, monograph on, terminating in impairment of mind.
—— decrease of, in the insane, 159.
—— hæmorrhage and softening of, dependent on congestion, 159.
—— overwrought, illustrations of, caution regarding, 289.
—— injurious effects of tight bandages on its functions, 37, 38.
—— irritation of, in children, 43.
—— symptoms of, 43.
—— treatment of, 43.
—— phantoms of, described and analized, 273—275.
——————— Abercrombie on, 275.
Brougham, Lord, on partial insanity, 323.
Bull of Innocent VIII. against demon worshippers, 229.
Burns, peculiarities of, 280.
—— theory of the poetic temperament of, 281.
Bush, Dr., on juvenile delinquency, and degeneration in upper classes of society, 428.
Byron, traits of his impetuous character, 279
Cairo, Grand, barbarous treatment of lunatics at, 243, 244.
Calmeil on mental epidemics, 221.
——— History of dæmonomania, chorea, monomania religiosa, 135.
Cambrai, nuns of, demonopathy among, 230.
Camisole, sufficient in general as a means of restraint, 192.
Canada, Upper and Lower, great disproportion between idiots and insane, 142.
Cannabis Indica, action of, 171.
Cannibalism of sorcerers, theory of its origin, 228.
Caribbees, alter the form of the cranium by pressure, 34.
Carlini, anecdote of, 289.
Carotids, compression of, in insanity, 144.
Carus, Dr., on the pantheistical theory of life, 68.
Cases illustrative of transient insanity, 330.
—— illustrating the effects of restraint, 260, 261.
—— of Tartini, 268.
—— of Tasso, 267.
—— of Wollaston, by Dr. Holland, 270.
Causes of death, table of, 220.
Catheterism, esophageal, of insane, 156.
Cephalœa, intermittent, dependent on cerebral effusion, 170.
Cerebral diseases of children, 41.
——— affections in children, obscure character of, 41.
——— affections often latent, 41.
——— affections of infancy and childhood, by Dr. Duke, 41.
——— congestions, chemical indications of, from blood, 122.
Changes during periods of adolescence in males and females, 5.
Characteristics and pathology of insanity 539.
Chemistry, human, pathological, and comparative, discordance between, 124.
Cherokees, insanity among, 143.
Cheromania, what, 271.
———— illustration of, 520, 521.
———— phenomena of, totally irrespective of constitutional disturbance, 274.
Cheyne, Dr., of Dublin, his description of acute hydrocephalus, 47.
Children, cerebal diseases of, 41.
Chloroform in tetanus, 168.
———— fractional doses of, 347.
Cholera, fear of, cause of insanity, 214.
Chorea in scrofulous subjects, 171.
Chronic hydrocephalus, 56.
Circulation, obstructed, effects on sensation, 91.

Classes, upper, of society, juvenile delinquency, and degeneration in, by Dr. Bush, 428.
Classification of mental diseases according to Brierre de Boismont, 551.
Clergymen, whether catholics or protestants, to be attached to every lunatic asylum in Ireland, 397.
——— of Cupar, singular *divining* anecdote of, 522.
Climateric, grand, 78.
Cold bathing and urtication successfully employed in palsy, 168.
Cold affusion, dangerous consequences from, 165.
Coleridge, his theory of life, 58.
———'s early life disclosed his metaphysical tendencies, 66.
——— influence of German literature upon his mind, 66.
——— peculiar character of his metaphysical philosophy, 6.
——— his theory truly pantheistical, 67.
——— argues physical properties of matter as essentially vital, 68.
——— opposes idea of life, considered as the mere result of organization, 69.
Commissions in lunacy, 468.
Compression of skull by bandages produces idiotism, according to M. Virey, 34.
Condition, married, strictly consonant with nature, 6.
Confidence, influence on the mind, 94.
Confinement, solitary, effect of, on the mind, by Dr. Winslow, 115.
——— injurious to the mind, without inducing insanity, 115.
Congestion of brain rare in early life, 42.
——— symptoms of, 42.
——— rapidity of their progress, 42.
——— treatment of, 42.
Congestion, cerebral, its connexion with hæmorrhage and softening of the brain, 159.
——— general indications of cerebral, corresponding with chemical analysis of the blood, 122.
Congress of science in Italy, subjects proposed at, 136.
Consciousness, double case of, 528.
——— connected with hysteria, 456.
Connexion between physiology, psychology, natural theology, and other sciences, 309.
Conolly, on accommodation required for lunatics capable of paying a small sum, 394.
Conolly, uncompromising advocate for non-restraint system, 254.
Constantinople, description of lunatic asylum at, 244.
Convictions, intuitive, proof of, 312.
Convulsions in children, 44.
Copland, Dr. James, on the young sleeping with the aged as a cause of depressed vitality, 74.
Cornwall, divining rod in, 515.
Correspondence from Paris, 338—348.
Cranium, deformed and imperfect intellect, 36.
Crania, infantile, on deformity of, 30.
——— modified forms of, from tight bandaging, illustrated by plates, 32, 33, 39.
Criminal acts, *prima facie* insane, 117.
——— illustrated by analogy, 117.
Cowper, his religious melancholy, 284.
Cure, period of life at which most common, 219.
—— time required for, in insanity, erroneous notion of the public, 218.
—— time required for in, insanity, 218.

D'Alembert, anecdote of, on his death bed, 89.
Dangerous influence of imagination on reason, Hume on, 61.
Davy, Dr. John, account of lunatic asylum at Constantinople, 244.
Death, allegorical conversation with, 89.
—— or dying, difference between, and going to sleep, 79.
—— accidental, 78.
—— apparent, 162.
—— bed scenes portrayed, 81.
—— bed interview of Stella with Dean Swift, 363.
—— fetch, story of, 521.
—— reasons for intimating to patient, 87.
—— resuscitation from, 79.
—— scene on the battle field, 82.
—— phenomena of, 76.
—— successive, of the different ages in individuals, 77.
—— of St. Laurence Justinian, 82.
—— lucid interval before, 83.
—— peculiarities of, when brain dies first, 84.
—— time of, often foreseen and prepared for, 85.
—— physical description of, leading features of, 85.
—— reflections on, what is the precise duty of the physician before the closing scene, 88.
—— should it be intimated to the patient? 86.

Death, ubiquity of, 77.
—— wary, anecdote of rational old lady thereon, 87.
—— why it is expedient to interdict the idea of, from the sick chamber, 86.
—— of Dr. Prichard, 188.
Decrease of brain in insanity, 159.
Definitions, preliminary, not essential, Whewell on, 68.
Deformity of infantile crania, 30.
—— crania, relative proportion in the sexes, 35.
Delany, Dr., account of confirmed maniacal state of Dean Swift, 367.
Delaye, Dr., on the peaked heads of the natives of Toulouse, 36.
Delirium, acute, of the insane, 552.
—— electrical, theory of, by Leopold Turk, 137.
—— tremens, tabular view of cases in Bloomingdale Asylum, 193.
—— classes most subject to, 193.
—— cases of, table of ages of, 193.
—— civil condition, table of patients, 194.
—— all cases, table of results of first admission, 194.
—— fatal cases, tables of crises in, 195.
—— leading features of malady described, 195—197.
—— general hopeless character of cases of, 197.
—— treatment of, 165.
Demon worshippers, bull of Innocent VIII. against, 229.
Demonomania, prevalent in fifteenth century, history of—horrors of, glanced at, 237.
—— historical and pathological account of, 134.
Description of spermatoza, 17.
Difficulties, pecuniary, potent cause of insanity, 211.
Disease, physical expression of, 2, 3.
—— sleeplessness in, 373.
—— influence on temper, 3.
Diseases, mental, classification of, according to Brierre de Boismont, 551.
—— mental, study of, 174, 495.
Divining rod in Cornwall, 515.
Domestic troubles, causes of insanity, 214.
Doses, fractional, of chloroform, 347.
Droin, Marie Madeline, medico-legal inquiry into state of her mind, 157.
Dryden's opinion of Swift, 351.
Dudley, Lord, traits of his character, 280.
Duke, Dr., on the cerebral affections of infancy and childhood, 41.
Dying, phantasies of the, not to be slighted, 82.
Dyspepsia, a cause of insanity, 210.

Earle, Dr., history of Bloomingdale Asylum, New York, 189.
—— M. J. W., new exposition of functions of nerves, 302.
Eclampsia, cured by stramonium, 172.
Education, errors in, productive of insanity, 214.
Edilin, Dr., of the Sorbonne, first open preacher against sorcery, 228.
—— his punishment, 228.
Ehrenberg, denies any unequivocal instance of spontaneous generation, 72.
Electro-biology, Smee on, 292.
Emissions, solitary, various conditions under which they occur, 21.
Emotional, state of the mind, during intellectual exertion, 286.
Employment, want of, cause of insanity, 211.
Engelken, water cure and electricity recommended by, in insanity, 137.
Encephalitis, treated by mercurial blisters, 170.
Epidemics, mental, by Dr. Calmeil, 221.
Epilepsy, treatment of, 144.
—— by Mettais and Breton, 154.
Equalization, principle of, in nature, 13, 14.
Erethism of brain in infants—*vide* Brain, irritation of.
—— of mind, 281.
Erlenmeyer, on the blood of the insane, 139.
Esquirol, rates of mortality by, in different forms of mental diseases, 410.
—— his classification of insanity, 551.
—— on the relation of age to insanity, 543.
Ether, sulphuric, effects of inhaling, in insanity, 412, 413.
Exercise essentially requisite to healthy functions of mind, 116.
Exhumation of body of Dean Swift, 370.
Existence, of matter and mind, evidence of, rest on some ultimate principles, 312.

Facts, chemical, on the blood in the neuroses, 132.

Faculties, mental, influence on the condition of the body, 560.
Fallacies in experiments hitherto made in prosecuting the inquiry of the relation between nervous matter and galvanism, 558, 559.
Fear, a cause of insanity, 213—209.
Fever, followed by insanity, 213.
Fibrin, quantity of, in blood, in neuroses, 122.
Fichte, the fundamental principle of his philosophy, 65.
Fish, vitality of, after being congealed, 74.
Fifteenth century, extensive and gross delusions of, 229.
Foville, on deformity of crania in children, arising from head-dress, 30.
Fragments, psychological, 134.
French, absurd habit of, in compressing the head, 35.
——— employ personal restraint in insanity, 248.
Functions, bodily and moral, parallel between, 316.
——— reproductive, on psychology of, by Lallemand, 1.

Galvanism and nervous influence not identical, 302.
——— fallacies in experiments hitherto made, respecting their identity, 558, 559.
Gambérini, on nocturnal neuralgia of fore-arm, 155.
Gastralgia and hypochondriasis, 165.
Generation, spontaneous, no proof thereof, 72.
Genius, enthusiasm of, to be kept down in early life, 281.
——— eccentricities of, 265.
——— irritability of, 276.
——— men of, the insanity of, 279.
——— early germs of, 265.
——— mortality of, 277.
——— its melancholy alliance with misfortune, 14.
Ghosts, genuine, 524.
German pantheism, 58—61.
Goëthe, wild dithyrambic of, on pantheism, 62.
Grief as a cause of insanity, 213.
Graves, Dr., on sleeplessness in disease, 378.

Hallucinations and illusions, Dr. Michea on, 138.
——— effects of, Calmeil on, 222.
——— of a homicide, singular mode of exposing, 235.
Hallucinations of genius, two modes or forms of, 271.
——— supposed connexion with excess, or deficiency of oxygenation of the system, 271.
Hamilton, account of form of Swift's skull, 371.
Hanwell asylum, deaths and injuries at, from disuse of restraint, 258.
——— lunatic asylum, fourth report of, 418.
——— male school of, described, 423.
——— history of individual scholars, 424.
——— female school, history of, 426.
——— individual scholars, their capacities and progress described, 426, 427.
Head of children, cautions as to tight caps, general mode of treating, 40.
Health, psychical, and disease, 139.
——— general ill, cause of insanity, 209.
Heberden, on lucid interval in phrensy, preceding death, 83.
Hegel, his pantheistical notions, 65.
Hereditary transmission of insanity, Dr. Earle on, 405.
Hohnbaum, on psychical health and disease, 139.
Holland, Dr., describes Dr. Wollaston's case, 270.
Home sickness, a cause of insanity, 213.
Homicidal insanity, tabular records of, 217.
Hope, influence of, on the body, 94.
Horne, Mr., essay upon madness, as treated by Shakspere, 589.
Horse, kick from, produces insanity, 207.
Human understanding, range of, 59.
Hume, on danger from imagination to reason, 61.
Hydrencephaloid disease of children described, 43.
——— symptoms of, and treatment, 43, 44.
Hydrocephalus, acute, treated by musk and blisters, 169.
——— or tubercular meningitis, description of, by Dr. Cheyne, of Dublin, 47.
——— connexion with strumous diathesis, 49.
——— early symptoms of, 49.
——— diagnosis of, 50—52.
——— prognosis of, 52.
——— morbid appearances peculiar to, 52, 53.
——— treatment of, 54.

Hydrocephalus, acute, prophylaxis in, 55.
———— chronic, 56.
———— morbid appearances, 57.
———— treatment of, 57, 58.
Hypochondriasis and gastralgia, 165.
Hysteria epileptiform, 160.
Hysteria, case of double consciousness, 456.

Ideler, Dr., influence of human science on psychology, 137.
Idiotism induced by compression of the skull, 34.
Idiots in Upper and Lower Canada, statistics of, 142.
—— school for, at Boston, 404.
—— in State of Massachussets, statistical account of, their capacities and conditions, 403.
—— school for, in Hanwell Lunatic asylum, 422—427.
—— in Massachussets, paternal regard of State for, 402.
—— successful management of, at Paris, 403.
—— wandering, great number of in Ireland, 393.
—— wandering, intermarriages of, 394.
Illusions and hallucinations, Dr. Michea on, 138.
——— of Benvenuto Cellini, 272.
——— and visions of Swedenborg, 273.
Illusionary character of mind of Shelley, 272.
Imagination, dangerous influence of on reason, remarks by Hume, 61.
Imbecility induced by solitary confinement, 116.
Impairment of mind, arising from softening of the brain, monograph on, by Dr. Winslow.
Impassioned letter of Vanessa to Swift, 359.
Incapacity, complete physical, unproductive of imaginary horrors, 12.
Inductions, pathogenic, founded on the chymical character of the blood in the neuroses, 133.
———— therapeutic, based on the same, 133.
Industry of the poets, 277.
Inequality and irrespondence of the sexual passions, typified in lays of troubadours and in song generally, 14.
Infanticide, 152
Infants, heads of, deformities of, induced by their coverings, observations of Dr. Foville on, 30.
Injuries, cases of insanity, arising from, 206.
Injury, severe bodily, cures a case of melancholy, 169.
——— brain of, specific gravity of, 538.
Insane, civil condition of, 201.
——— classification of, 551.
——— detention of, in gaols or workhouses, illegal in Maine District, United States, 410.
——— lectures to, on the physical sciences, 414.
——— number of, in Ireland, 392.
——— occupation of in life, tabular view of, 202.
——— occupation of females, tabular view of, 203.
——— œsophageal catheterism of, 156.
——— organization of, labour for the, 167.
——— proportion of the two sexes, 542.
——— pulse of, 143.
——— in Ireland, partly confined in gaols and cells of workhouses, 388.
————— insufficient accommodation for, 394.
————— pestilence unusually severe on, 393.
————— propositions submitted by House of Lords, for security and protection of, 389.
————— general condition of, 388.
——— palsy of, danger of repeated obstructions of blood in, 346.
——— on the use and abuse of restraint in the treatment of, by Hamilton Labatt, 240.
——— security they now enjoy, contrasted with former eras, 222.
Insanity, arising from cholera, fear of, 214.
———— difficulties, pecuniary, 211.
———— domestic troubles, 214.
———— dispepsia, 210.
———— exertion, excessive bodily, 207.
———— errors of education, 214.
———— fever, 209.
———— grief, 213.
———— health, general ill, 209.
———— mesmerism, 407.
———— prussic acid, 211.
———— religious causes, 212.
———— remorse, 213.

Insanity, arising from self-pollution, 207.
———————— shocks, mental, 214.
———————— study, excessive, 214.
———————— tobacco, 206.
——— connected with gout, 210.
———————— phthisis, 210.
———————— rheumatism, 210.
———————— rubeola, 210.
———————— scarlatina, 210.
——— America, in, 398.
——— Cherokees among, 143.
——— considered historically, theologically, judicially, and philosophically, by Calmeil, 221.
——— compression of carotids in, 144.
——— Dean Swift of, by Mr. Wilde, review of, 349.
——— delirium, not exclusive, test of, 338.
——— essential, nature of, 540.
——— hereditary, transmission of, Dr. Earle on, 405.
———————— predisposition to, 552.
——— different forms of, features of, 552.
———————— observations on, by Dr. Baillarger, 340, 341.
——— the, of men of genius, 262.
——— impulsive, 577.
——— lesions of intellect in, 545, 546.
——— motility, 549.
——— organic life, 550.
——— sensibility, 547—549.
——— incubation, period of, 545.
———————— state of functions during, 546.
——— mild treatment of, zealous efforts of Tuke to extend, 247.
——— mortality in, 410.
———————— ratio of, according to Esquirol, 410.
———————— Pinel, 410.
———————— Raymond, 410.
———————— Tenon, 410.
———————— of in northern states of America, 411.
———————— of, in Paris, in 1786, 410.
——— organic lesions, are they invariably present in ? 535.
———————— Esquirol, his opinion, 535.
———————— various opinions, 534-536.
——— pathological appearances in, 536, 537.
———————— Lelut, his opinions, 537, 538.
——— partial, Lord Brougham on, 323.
Insanity, pathology of, theories of, 251.
——— periods of life at which most prevalent, 200.
——— prevalent in fifteenth century, and its type, 223.
——— pathology and characteristics of, 534.
——— physical and moral causes of, by Dr. Earle, 405.
——— penitentiaries, influence of system of, in producing, 171, 341, 343.
——— plea of novel mode of procedure, in judicial cases, 408.
——— in plea of, surveillance proposed, with suggestions by the editor, 109.
——— plea of cases of, 331—333.
——— predisposition to, when greatest, 543, 544.
——— presentiment of, in Dean Swift, 364.
——— puerperal, 175.
———————— discussion on at Westminster Medical Society, 175.
——— ratio of in England, Scotland, Ireland, and Wales, data for not satisfactory, 394.
——— inspectors of, wide range of their duties, 395, 396.
———————— suggestions as to medical men, 397.
——— seasons of year, influence on, 544.
——— sedative treatment of, 319.
——— relative proportion of various forms of, 215.
——— suicidal, hereditary nature of, 554.
——— sulphuric ether, effects of inhalation in, 412.
——— spermatorrhœa, connexion with, 2.
——— stramonium, influence on, 342.
——— tobacco, soothing action of in, 406.
Intoxication, effects a cure on tetanus, 173.
Intuitive convictions, proofs of, 312.
Investigations, medico-legal, 344.
Irregularities of Dean Swift at college, 353, 354.
Irritability of genius, 276.
Isolation, early, high importance of, in insanity, 250.
Italy, scientific congress in subjects proposed at, 136.

Jacobi on the compression of the carotids in insanity, 144.
——— on the pulse of the insane, 143.

Jeffrey's remarks on Swift's conduct to Vanessa, 356.
Johnston, Samuel, religious monomania of, 282, 283.
Journal, British, of Psychological Medicine, by Dr. Winslow; encomium on by Sociéte Medico-Psychologique de Paris, 344.
Journal of Insanity, the American, 398.
Jurisprudence, medical, 331.
Judicial insanity, 630.
Junod, Dr., his exhausted air boot, 627.
——— in relation to insanity, 468.
Juvenile delinquency and degeneration in the upper classes of society, 428.

Kant, double origin of ideas, 64.

Labatt, on the use und abuse of restraint in the management of the insane, by Dr. Hamilton Labatt, 240, 262.
——— his opinion on restraint ambiguous, not sufficiently specific, 252.
——— general opinion of his work, 253.
Labour, organization of, for the insane, 167.
Lady, rational old, anecdote of, 87.
Lallemand, on spermatorrhœa, or the psychology of the reproductive functions, 1.
——— extreme, and widely extended range of subjects treated by him, 2.
——— importance of the work to society, 19.
——— of a purely physical character, 18.
——— honesty and originality of purpose of his work, 18.
Laws, physical, moral, and psychological, violation of, 20.
Lee on the nervous system, 302.
—— deductions of, in regard to the nerves, 308.
Lefevre, apology for the nerves, 90.
Lepois, gives a correct view of nervous disorders, 236.
Letters on the truths contained in popular superstitions, by Herbert Mayo, M.D , 512.
Life, definition of, by Smee, 292.
——— Bichat, 69.
——— Coleridge, 69.
—— only in vegetable and animal creation, 70.
—— in very simple forms of organization, 72.
—— ordinary duration of, 78.
—— theory of, 72, *et seq.*
—— on theory of developments in organization, 73.
Life, what it is, 68.
—— an independent principle, 72, 73.
—— and organization, Dr. Barclay on, 59.
—— principle of, distinct from that of soul and body, 74.
Lima, fashionable amusements at, 242.
Lincoln hospital for lunatics, first adopted the non-restraint as a system, 254.
Literary notice, 188.
Logic, its formulæ not suited to melancholic patients, 282.
Lunacy, law of, defective state of in Ireland, 397.
Lunatic asylums in America, 398.
——— in Ireland, Lords, House of, submit five propositions, for the protection and security of the insane there, 389.
Lunatics, lectures to, 414.
——— not criminal, committed to gaol in 1847-1848 in Ireland, 390.
——— insufficient accommodation for in Ireland, 394.
——— and wandering idiots in Ireland, intermarriages among, 394.
——— capable of paying a small sum, accommodation wanted for, 395.
——— law of, 178, 186.
——— amendments thereon, letter to Editor of Morning Chronicle, by Dr. Winslow, 182.
——— reply to, 185.
——— counter reply by Dr. Winslow, 186.
——— additional remarks of editor, exact nature of the question for discussion proposed, 187.
——— defective condition of, in olden times, 241.
——— revolution in management of, altogether of recent date, 241.
——— principle of restraint entirely to be discarded from, 249.

Madness very widely diffused according to popular language, 226, 264.
——— and reason not incompatible, 276.
Magnetism, animal, sympathy, the soul of, 519.
Male line, in, perfect forms of cerebral organization rarely transmitted, 13.
Mania, acute, 163.
——— *a potu*, Indians, exempt from, 416.
——— peculiar to English, Irish, and Germans, 416.
——— rare amongst Spanish, French, and negroes, 416.
Maine, (district of, in America), mode of determining the admission of supposed lunatics into asylnms, 408.

Maine, (district of, in America), important and judicious novel mode of procedure in cases of plea of insanity, 408.
——— general law against detention of insane in gaols or Houses of Correction, 410.
Massachussets, legislature of, grants 2500 dollars for experiments in training ten idiots, 404.
Marriage, less a physical impulse than a psychological relation, 7.
Married state, calls forth the exercise of self command, 7.
——— reflexions on, 6, 7.
Marriages amongst lunatics and wandering idiots in Ireland, 394.
Mayo, Dr., on popular superstitions, 512.
——— character of the work, 514.
Medical men, only to be appointed to Irish lunatic asylums, 397.
——— jurisprudence, 331.
Medico-legal investigation, 344.
Melancholy, cured by severe bodily injuries, 169.
Membranes false, of arachnoid, source of, 128.
——— not the result of sanguineous effusions, 129.
Memoirs on psychology, published on the continent in 1846, 1847, 134.
Men of genius, the insanity of, 262.
Meningitis, acute, tubercular in the adult, 168.
——— acute, rare in children, 45.
——— rapidly fatal, 45.
——— symptoms of, 45.
——— diagnosis of, 46.
——— treatment of, 46.
Mercurial blisters in encephalitis, 170.
Mesmerism, 527, 528.
——— a cause of insanity, 208.
——— characteristics of, 531.
——— brief statement of *modus operandi* of, 532.
——— probable good that may result from inquiries into, 533.
Mental diseases, pathology and therapeutics of, 134.
——— study of, 174, 495.
——— epidemics, by Calmeil, 221.
——— diseases, morbid anatomy of, 483.
——— symptoms of spermatorrhœa, 18.
Metaphysical and physical conditions mutually opposed, 13.
M'Dougall's translation of Lallemand on spermatorrhœa, 1.
Michea, Dr., on the blood as the neuroses, 118.
——— on hallucinations and illusions, 138.
Mind, erethism of, 281.
——— effects of solitary confinement on, by Dr. Winslow, 115.
——— evidence for, stronger than that for matter, 314.
——— existence of in lower animals, 314.
——— principle of, distinct from that of life, 76.
——— and matter, belief of existence of, rest on same ultimate principles, 312.
——— immateriality of, existence of the Deity, and vital principles, theory of, closely interwoven, 59.
——— impairment of, from softening of the brain, monograph on, by Dr. Winslow, .
Miscarriages, causes of occasional, in the lower orders, 9.
Months, admission for insanity, according to, in Bloomingdale Asylum, 198.
Moral laws, violation of, 20.
Morell's reflections on ideas attached to Spinoza, theory of a first cause, 64.
Mortality in insanity, 410.
——— mania, 410.
——— monomania, 410.
——— lypomania, 410.
——— in dementia, 410.
——— ratio of in Northern State of America, 411.
——— of insane in Paris, 1786, 410.
——— peculiarly great amongst insane during Irish pestilence, 393.
Motiveless, crime of the young, Dr. Wigan on, 499.
Mulattoes, or hybrid race, characteristics of, 415.
——— differences between, as of Spanish or French extraction with negro, and Anglo-Saxon, 415.
Murder, attempt of insane to commit, 152.
Museums for the insane, 414.
Muscular excitability dependent on nerves, 305.
Music, its effects on the nerves, 173.
Musk and blisters in acute hydrocephalus, 169.

Nantes, lunatic asylum at, facts connected with its history, 147.
Nature, general principle of, philosophy of, by Stallo, 58.

Nature, animated, the philosophy of, or the power and action of the nervous system, by G. Calvert Holland, M.D., 555.
——— character of the work, 555.
Nervous affections, periodic, cured by sulphate of quina, 171.
Nerves, apology for, by Sir George Lefevre, 90.
Nervous system, the, 302.
——— brain, the centre of, 302.
Nerves, association of, 306.
——— classification of, by Mr. Earle, 303.
——— deductions regarding, by Mr. Lee, 308.
——— exposition, new, of their functions, by Mr. Earle, 302.
Nervous influence, not identical with galvanism, 302.
——— principle, the, and discovery of the circulation of the blood, compared and contrasted, 555.
——— power, circulating theory of, by Mr. Earle, 305.
——— respiratory, tract of Sir Charles Bell, called in question, 306.
——— secondary relations of highest importance in pathological questions, 90.
——— system, sympathetic nerve, appendage of, 307.
Nerves and nervous maladies, pathology of, by Sir George Lefevre, 90.
Neuroses, on the blood in, 118.
Nomenclature of insanity, extreme difficulty of, 215.
Nostalgia, a cause of insanity, 213.
Nott, Dr., characteristics of white negroes and mulattoes, 415.
Novel reading, three cases of insanity from, 215.
Nottidge *versus* Ripley, trial of, 630.
Nurses, vile and vicious practices of, 26.
Nymphomania, cured by tartar emetic ointment, 141.

Objections to effects of pressure on circulation within the brain, 38.
Opinions on origin of false membranes in arachnoid, 128.
Opium, in sedative treatment of insanity, 320.
——— cases of mania in which it should be given, 320.
Orchideal discharge, and sexual, effects contrasted, 22.
Organization, not the cause of life, according to Coleridge, 69.
——— illustrated, 69.
Origin of Swift's vertigo accounted for, by Dr. Beddoes, 354.
Orleans, New, description of Charity Hospital there, 416.
——— frequency of mania *a potu*, in the lunatic department there, 416.
Orrery's Lord, remarks on Swift's anticipation of his lunacy, 364.
——— his description of intellectual character of Stella, 357.

Padded room for the insane, 259.
Paganini, his talent, and what it cost him, 278.
Palsy, general, in the insane, remarks on, 411.
——— less prevalent in America than in Europe, 412.
——— on the increase, 412.
——— more common in men than women, 411.
——— dangers attending oft-repeated abstractions of blood in, 346.
Pantheism, German, 58.
——— of ancients and Germans, 61.
——— of philosophy and poetry distinguished, 62.
Paralysis, idiopathic, 155.
——— of tongue cured by galvano-puncture, 162.
——— complete, cured by cold-bathing and urtication, 168.
Partial insanity, Lord Brougham on, 323.
Paris, correspondence from, 338, 624.
——— insane of, mortality in 1786, 410.
Pathology of nerves and nervous maladies, Sir George Lefevre on, 90.
——— and characteristics of insanity, 534.
——— of insanity, theories on, 251.
——— and therapeutics of mental diseases, 134.
Pathological human chemistry, and comparative animal chemistry, discordance between, 124.
Peaked head, cause of, in south of France, 36.
Pecuniary difficulties, cause of insanity, 211.
Penitentiary system, influence of on insanity, 171.
Pestilence and famine affected the insane with peculiar severity, 393.
Phantasies of the dying not created in vain, 82.
——— shadow forth the world to come, 82.
——— of a haunted man, 269.
Phenomena of death, 76.

Philosophy, the, of animated nature, on the laws and actions of the nervous system, by Dr. G. C. Holland, 555.
Phlebitis in puerperal women, 153.
Phrenology, fallacies of, 539.
Physical and metaphysical sciences, 309.
——— laws, violation of, 20.
——— properties of matter essentially vital, according to Coleridge, 68.
——— and metaphysical conditions mutually opposed, 13.
Physiology, natural theology, and other sciences, the relation between, 309.
——— intermediate between physical and metaphysical sciences, 309.
Picture, dreadful, of present state of lunatics in Ireland, 391.
Pinel, triumphant success of, removing the fetters from the insane, 247.
——— rate of mortality by, from insanity, 410.
Plea of insanity, 331.
——— novel and important mode of precedure in judicial cases, 408.
——— surveillance proposed in cases of, and suggestions by the editor, 409.
Poetic character close relation to the insane state, 262.
Pollock, Chief Baron, comments on his observations relative to laws of lunacy, 564.
Pomade of the sorcerers, 231.
Ponzinibius advocates the theory of errors of the senses, in opposition to the belief in demons, 234.
Possession, supposed demonical, described by the present sufferer, 462.
Power of organism not fixed, 557.
Presentiment of insanity in Swift, 364.
Pressure, injurious effects of external pressure on the brain, 37, 38.
——— never to be applied to head of newly born infant, 39.
Pritchard, Dr., death of, 188.
Principle of life, distinct from that of mind or soul, 76.
Proportions of the two sexes in insanity, 542.
Prussic acid, case of insanity from, 211.
Psychical health and disease, in their various transitions, 139.
Psychology relative to physiology, natural theology, &c., 309.
——— important works and memoirs on, published on the Continent in 1846-47, 134.
Psychological correspondence from Paris, 624.
——— effects of spermatorrhœa, 18.
Pulse of the insane, 143.
Punishment, corporal, for insanity, employed in Grand Cairo, 244.

Raisonnante, La Folie, interest connected with, 276.
Ratio, between males and females in Bloomingdale Asylum, 200.
Re-action, in favour of non-restraint system, theory of, 246.
Reason and madness not always incompatible, 276.
Reichenbach, explanations offered by, of mysterious coincidences, 523.
Reid, Dr. James, on the nervous system and nervous disorders, 281.
Reflexions on married state, 6, 7.
Relation, subsisting between degree of deformity of cranium and imperfect state of intellect, 36.
——— of thought to matter, 556.
Religion, as a moral course of insanity, 212.
Religious opinions, character of, modify forms of insanity, 224—225.
Remorse, insanity from, 213.
Renovation of body at climateric periods, 78.
Report of inspectors of lunatic asylums in Ireland, melancholy extract from, 390, 391.
Reproductive functions, physiology of, by Lallemand, 1.
Restraint, on the use and abuse of, in the treatment of the insane, by Dr. Labatt, 240.
Restraint, little, employed in Irish asylums, 256.
——— only recently removed on general principles, 245.
——— triumphant success of Pinel in removing, 247.
——— personal, still employed by the French, 248.
——— non, system of, introduced at Lincoln Lunatic Asylum, 254.
——— great advocate for, Dr. Conolly, 254.
——— completely rejected at Retreat of Friends, at York, in 1843, 255.
——— camisole generally adequate in, 192.
——— rational, view of, and reasonable objections to absolute non-restraint, by managers of Bloomingdale asylum, 192.
Resusitcation from death, instances of, 79.
Retirement from active life, noxious effects of, 117.

Rheumatism and gout, effects of, in producing insanity, 210.
Rive, de la of Geneva, apopthegm, on treatment of the insane, 246.
Roger's account of mal-practices in mad houses, referred to, 247.
Rousseau, a victim of self-indulgence, 15.
——— confesses to self-abuse on philosophical (?) principles, 16.
——— Lallemand, his true commentator, 27.
Rubeola, in connexion with insanity, 210.
Rush, Dr., opinion of, liability of sexes to insanity, 211.

Sale (of Koran Fame), anecdote of, 30.
Stallo, general principles of philosophy of, 58.
—— unquestioned mysticism of the work, 67.
Sanity of mind of Swift argued erroneously by Mr. Wilde, 366.
School for idiots at Boston, 404.
Schelling, his elementary metaphysics, 65.
Schnitzer, Dr., on pathology and therapeutics of mental diseases, 134.
Scott, Sir Walter, memory, the source of his fame, 290.
——— on personal appearance of Stella, 357.
Scott, Sir Walter, and the poets contrasted, 278.
Seasons, influence of, in production of insanity, 199.
Seclusion, in darkened apartments, in certain cases of insanity advised, 259.
Sedative treatment of insanity, 319.
Seeds, remarkable vitality of, 74.
Seer gift, Mayo on, 525.
Senses, transference of, 527,
Selections, 143, 345.
Self-abuse, state of mind induced by described by a sufferer, 27.
Self-reproach, natural result of self-abuse, 26.
Separation from home and home scenes, absolute in cases of insanity, 249, 250.
Serum, amount of in blood of the neuroses, 122.
Shakespeare, cause of his death, 290.
——— the successive seven ages of, death of, in each individual, 77.
——— madness as treated by, 3.
Shelley, the poet, illusions of, 272.
Sheridan, Dr., describes interview between Swift and Stella on her deathbed, 363.
Shock, mental, insanity from, 214.
Show-rooms, lunatic asylums formerly used as such, 242.
Site of Bloomingdale asylum described, 191.
Sleep, reflexions on, 378.
—— exhausting influence of want of, 208.
—— loss of, with excessive bodily exertion inducing insanity, 207.
—— and dying, distinction between, 79.
—— writing, 530.
Smee, definition of life by, 292.
—— on electro-biology, 292.
Society, medico-psychologique de Paris, acknowledges the British Psychological Journal, 344.
Socrates, his demon, 273.
Solitary confinement, effect of, on the mind, by Dr. Winslow, 115.
Solitude, a desire for to, be looked on with suspicion, 28.
Sonata, Devil's, in what state composed, 268.
Song, the natural outburst of misplaced and hapless affections, 14.
Sorcery, referred to disorders of the intellect, 234.
——— its misdeeds in the fifteenth century, 226.
——— its diffusion and progress, 227, 228.
Sorcerers, their pomade, 231.
——— identity of mental character of, with modern hallucinations of the insane, 227.
Soul, distinct from either life or mind, 76.
Sounds and voices, real, heard by reputed demons, 233.
Specimen of range of duties attached to the office of inspector in Ireland, 395, 396.
Spermatorrhea, medical treatise on, by Lallemand, 1.
——— apology for consideration of, founded in false delicacy, 1.
——— insanity traced to, 2.
——— moral symptoms of, 18.
——— origin of, average of cases, 23.
——— natural causes of, and checks to, 23, 24.
——— danger of, unduly magnified by quacks and pretenders, 24.
——— bad consequences from trifling with, 24.
——— endless frauds of designing empirics in, 24.
——— sometimes vicarious with cuticular disease, 25.
——— cure of, by local application of nitrate of silver, 25.
——— psychological effects of, 25.

Spermatorrhœa, induced by ascarides, 26.
——— physical causes of, 26.
——— physical effects of comparatively trivial, 29.
——— psychological consequences more to be apprehended, 29.
——— a distinct monomania, a "true thing," 30.
Spermatozoa, description of, 17.
Spinoza, his philosopy and its effects, 63.
——— reflections of Morell on his interpretation of the word "God," 64.
Spirit, philosophical of modern treatment of the insane, 259.
Statistics of insanity, New York, 1821, 1844, 1847, 190.
——— and history of Bloomingdale asylum, New York, 189.
——— system of treatment adopted there, 191.
Statistical account of the condition and capacities of idiots in Massachusetts, 403.
——— tables of relative proportions of deformed head in the lunatic asylum of the Lower Seine, 35.
——— tables of poor lunatics in Ireland, 392.
Stella, her dying request to Swift, 363.
——— character of, by Lord Orrery, 356.
——— personal appearance of, by Sir Walter Scott, 357.
Stramonium, action of, in the insane state, 342.
——— effects the cure of eclampsia, 172.
Study, intense, a cause of insanity, 214.
——— excessive, bad effects of, 288.
——— of mental diseases, 174.
Suicides in France, 347.
——— peculiarities in character of, minds disposed to, 216.
——— persons who threaten never commit, 216.
——— cunning concealment in those predisposed to, 216.
Sulphuric ether, effects of inhalation of, in cases of insanity, 412.
Sumner, account of amelioration of idiots in France, 403.
Superstitions, popular, letters on the truths contained in, by Herbert Mayo, M.D., 512.
——— among mankind, reflections on, 512—514.
Surveillance in cases of plea of supposed insanity proposed, with reasons thereon, by the Editor 409.
Swedenborg, visions of, 273.
Swift, dean, melancholy presentiment of his insanity, 364.
——— remarks on, by Lord Orrery, 364.
——— confirmed madness of, pourtrayed, 367.
——— insanity of, 349.
——— apology for, 350.
——— brief estimate of his character as a literary writer and poet, 351.
——— unsuccessful effort of Mr. Wilde to establish his sanity of mind, 366.
——— post-mortem examination of, 369.
——— exhumation of body of, 370.
Sympathetic system, an appendage of the nervous, 307.
Sympathy, the soul of animal magnetism, 519.
System, the nervous, 303.

Tables, statistical, of poor lunatics in Ireland, 392.
——— of lunatics in private asylums, 392.
——— of wandering idiots, 392.
Tartar emetic ointment in nymphomania, 141.
Tartini, reverie of, 268.
Tasso, vision of, 267.
Taylor, anecdote of, 30.
Tetanus cured by intoxication, 173.
——— chloroform in, 168.
Theomania of Jeanne d'Arc, 225.
Theologians and surgeons, apparently leagued together in preceding centuries, 232.
Theology, natural, the connexion between, physiology, psychology, and the sciences, 309.
Theory of life, 58.
Theories of pathology of insanity, 251.
Therapeutics and pathology of mental diseases, 134.
Tobacco, use of, in insanity, 205.
——— soothing effects of, in insanity, 406.
——— supposed cause of insanity, 406
Toledo, lunatic asylum of, described, 242.
Tongue, palsy of, cured by galvanopuncture, 162.
Toralba, curious case of, 239.
Toulouse, prevalence of peaked heads at, 36.
Transient insanity, 329.
——— Spectator, (newspaper), on 329.

Transmission, hereditary, of insanity, Dr. Eade on, 405.
——— of most perfect forms of cerebral organization, seldom occur in male line, 13.
Travelling commission of managers of New York State Lunatic Asylum, interesting extracts from report of, 413
Treatment, sedative, of insanity, 319.
Troubadours, their songs typify the inequalities of the sexual relations, 14.
Tubercular meningitis, *vide* acute hydrocephalus, 47.
Tuke, his exertions in forwarding the mild treatment of insanity, 247.
Turk, the electrical theory of delirium, 137.

Ulvas, (conferva), certain singular products of, observed by Bory de St. Vincent, 71.
University, Swift's equivocal career at, and degradation, 353, 354.

Vampyres, what they *are* and *do*, 517
——— career of, by Mayo, 517—519.
Vampire, the French, 577.
Vanessa, impassioned letter of, to Swift, 359.
Varina, (Jane Waring), letter of Swift to, 355.
——— Jeffrey's remarks on Swift's conduct to her, 356.
Various conditions under which solitary emissions take place, 21.
Vision of Tasso, 267.
Vital power depressed, from young sleeping with the aged, 74.
——— principle, supposed site of, 75.
——— proof of existence from its effects, 76.
——— opposed to usual actions of physical agents on organic functions, 75.
——— distinct from soul and mind, 76.
——— properties not dissimilar to physical, according to Coleridge, 68.
Vitality of seeds, 74.
——— of fish, after being congealed by cold, 74.
Voltaic battery, existence of in animal body, 293.
Voltaire on demoniacs, 238.

Wandering idiots, excessive number of, in Ireland, 393.
Ward, Dr., case of double-consciousness, 456.
Waywardness of Swift, anecdote of, 365.
Webster, Dr., remarks of, prevalence of suicide during system of restraint in insanity, 257.
——— on puerperal insanity, 175.
Whewell, Professor, denies definitions necessarily precede conceptions, 68.
Wigan, Dr., the unpublished MSS. of, 497.
——— character of, 498.
——— nature of the MSS., 499.
——— on motiveless crime of the young, 499.
——— on the physical character of those persons, 500,
——— on the change in the contour of the bones of cranium and face, about the age of 16, 501.
——— theoretical view of the cause of the motiveless crimes, 502.
——— treatment of, 502.
——— on spasm of the nervous fibre, 504.
——— on Heinroth's hypothesis, 504, 505.
——— on senile dementia, 505, 506.
——— on anxiety, 507—510.
——— on analogy of mind of insane and children, 511.
——— anecdote by, of nursery, for the instruction of all, 511, 512.
——— phantasms of a haunted man, 269.
Wilde, Mr., argues the sanity of Dean Swift, 366.
Winslow, Dr., on the effect of solitary confinement on the mind, 115.
Women, puerperal, phlebitis in, 153.
Works, important, and memoirs on psychology, published on the Continent, in 1846-47, 134.

York, New, Bloomingdale Asylum at, 189

Zschokke, the divining power, 525, 526.

